# Naturally occurring mutations in PB1 affect influenza A virus replication fidelity, virulence, and adaptability

**DOI:** 10.1186/s12929-019-0547-4

**Published:** 2019-07-31

**Authors:** Ruey-Wen Lin, Guang-Wu Chen, Hsiang-Hsuan Sung, Ren-Jye Lin, Li-Chen Yen, Yu-Ling Tseng, Yung-Kun Chang, Shu-Pei Lien, Shin-Ru Shih, Ching-Len Liao

**Affiliations:** 1 Graduate Institute of Life Sciences, National Defense Medical Center, No. 161 Section 6, Minquan E. Road, Taipei, 114 Taiwan; 2 Research Center for Emerging Viral Infections, College of Medicine, Chang Gung University, No. 259, Wen Hwa 1st Road, Kwei-Shan, Taoyuan, 333 Taiwan; 3 Department of Laboratory Medicine, Linkou Chang Gung Memorial Hospital, No. 5 Fu Hsing Street, Kwei-Shan, Taoyuan, 333 Taiwan; 4 Department of Computer Science and Information Engineering, School of Electrical and Computer Engineering, College of Engineering, Chang Gung University, No. 259, Wen Hwa 1st Road, Kwei-Shan, Taoyuan, 333 Taiwan; 5 National Laboratory Animal Center, Nation Applied Research Laboratory, No.106, Sec. 2, Heping E. Rd., Taipei, 10622 Taiwan; 6 National Mosquito-Borne Diseases Control Research Center, National Health Research Institute, 10 F, Bldg F, 3 Yuanqu Street, Taipei, 11503 Taiwan; 7 Department of Microbiology and Immunology, National Defense Medical Center, No. 161 Section 6, Ming Chaun E. Road, Taipei, 114 Taiwan; 8 National institute of Infectious Diseases and Vaccinology, National Health Research Institutes, No. 35, Keyan Road, Zhunan, Miaoli County 35053 Taiwan; 9 Graduate Institute of Biomedical Sciences, Department of Medical Biotechnology and Laboratory Science, College of Medicine, Chang Gung University, No. 259, Wen Hwa 1st Road, Kwei-Shan, Taoyuan, 333 Taiwan

**Keywords:** Influenza A/H1N1, PB1, RdRp, Fidelity, Fitness, Neuraminidase

## Abstract

**Background:**

Mutations in the PB1 subunit of RNA-dependent RNA polymerase (RdRp) of influenza A virus can affect replication fidelity. Before the influenza A/H1N1 pandemic in 2009, most human influenza A/H1N1 viruses contained the avian-associated residue, serine, at position 216 in PB1. However, near the onset of the 2009 pandemic, human viruses began to acquire the mammalian-associated residue, glycine, at PB1–216, and PB1–216G became predominant in human viruses thereafter.

**Methods:**

Using entropy-based analysis algorithm, we have previously identified several host-specific amino-acid signatures that separated avian and swine viruses from human influenza viruses. The presence of these host-specific signatures in human influenza A/H1N1 viruses suggested that these mutations were the result of adaptive genetic evolution that enabled these influenza viruses to circumvent host barriers, which resulted in cross-species transmission. We investigated the biological impact of this natural avian-to-mammalian signature substitution at PB1–216 in human influenza A/H1N1 viruses.

**Results:**

We found that PB1–216G viruses had greater mutation potential, and were more sensitive to ribavirin than PB1–216S viruses. In oseltamivir-treated HEK293 cells, PB1–216G viruses generated mutations in viral neuraminidase at a higher rate than PB1–216S viruses. By contrast, PB1–216S viruses were more virulent in mice than PB1–216G viruses. These results suggest that the PB1-S216G substitution enhances viral epidemiological fitness by increasing the frequency of adaptive mutations in human influenza A/H1N1 viruses.

**Conclusions:**

Our results thus suggest that the increased adaptability and epidemiological fitness of naturally arising human PB1–216G viruses, which have a canonical low-fidelity replicase, were the biological mechanisms underlying the replacement of PB1–216S viruses with a high-fidelity replicase following the emergence of pdmH1N1. We think that continued surveillance of such naturally occurring PB1–216 variants among others is warranted to assess the potential impact of changes in RdRp fidelity on the adaptability and epidemiological fitness of human A/H1N1 influenza viruses.

**Electronic supplementary material:**

The online version of this article (10.1186/s12929-019-0547-4) contains supplementary material, which is available to authorized users.

## Background

The genome of influenza A viruses (Family: *Orthomyxoviridae*) contains eight segments of single-stranded, negative-sense RNA. Antigenic shift results from the reassortment of genomic segments from different strains of influenza A viruses, often from different host species. The unique antigenicity of these newly emerging reassortant strains can evade existing herd immunity against circulating seasonal influenza A viruses, and this type of punctuated antigenic variation has contributed to influenza pandemics throughout history. As a pandemic influenza strain becomes the most prevalent influenza virus in the population, it contributes its unique genetic characteristics to the gene pool of subsequent seasonal influenza viruses.

Since its emergence in early 2009, the swine-origin pandemic 2009 influenza A/H1N1 virus (pdmH1N1) has become a circulating seasonal human influenza virus. Despite its temporal association with swine A/H1N1 [1], the pdmH1N1 genome contains multiple reassortant viral genes derived from avian influenza viruses [2]. The PB2 and PA genomic segments of pdmH1N1 originated from an avian influenza virus that had previously reassorted into a swine influenza virus in 1998 [3]. The PB1 genomic segment of pdmH1N1 was recently acquired from a human seasonal influenza A/H3N2 virus, which had previously acquired PB1 from an avian influenza virus in 1968 [4]. The hemagglutinin (HA), nucleoprotein (NP), and nonstructural (NS) genomic segments of pdmH1N1 are from a North American swine influenza virus lineage that can be traced to the pandemic 1918 A/H1N1 virus [5], and the neuraminidase (NA) and matrix (M) genomic segments are from a Eurasian swine virus that previously acquired both segments from an avian influenza virus in 1979 [6, 7]. The overall influence of the emergence of pdmH1N1 on the gene pool of currently circulating seasonal human influenza viruses remains largely unclear.

Like most RNA viruses, the RNA-dependent RNA polymerase (RdRp) of influenza viruses has a higher error rate than that of DNA polymerases because it lacks a proofreading mechanism [8–10]. Nucleotide misincorporation by RdRp during replication contributes to antigenic drift, which increases the probability of the virus evading host immunity against seasonal influenza viruses. According to the quasispecies theory, the inherent infidelity of RdRp drives the formation of variant clouds in the influenza virus population that consist of diverse genetic variants which are linked through shared mutations. These variants collectively contribute their antigenic characteristics to the influenza virus population, and interact cooperatively at the functional level as selective pressure acts upon the population as a whole [11]. Mutant clouds provide dynamic repositories of variants permitting certain viruses to undergo adaptation to selective pressures, including species barriers, host immune responses, and antiviral agents. An increased mutation rate allows RNA viruses more opportunities to adjust to environmental stresses, whereas elevated RNA replicase fidelity, despite allowing a virus to stably pass on its genetic traits to its progeny, actually serves to restrict genetic diversity among the viruses that occupy the greatest space in the fitness landscape.

Fidelity determinants for an RdRp were first described for poliovirus [12–14] and chikungunya virus [15], both of which are single-stranded, positive-sense RNA viruses. Variant viruses of each exhibited a certain degree of attenuation or fitness loss in vivo, which was likely the result of restricting genetic diversity at the cost of increasing fidelity. Site-directed mutagenesis of predicted key residues in the RdRp of Coxsackie virus B3 [16] and the exoribonuclease of coronavirus [17, 18] produced some variants with mutator phenotypes that were less virulent in vivo than the wild-type parent viruses. Altering the fidelity of RdRp clearly affects the virulence and fitness of RNA viruses in vivo, demonstrating the critical role that RdRp plays in balancing pathogenesis with adaptation.

By a large-scale, entropy-based computational algorithm of influenza A virus sequences deposited in the Influenza Virus Database (https://www.ncbi.nlm.nih.gov/genomes/FLU/ Database/nph-select.cgi?go = database), which is primarily a standard prevalence/frequency analysis, we have previously characterized avian- and human-specific genomic signatures [19], which showed that most avian viruses contained serine at PB1–216 (96.6%) and valine at PB1–336 (98.8%), whereas all of the pdmH1N1 sequences contained glycine at PB1–216 and isoleucine at PB1–336. PB1–216 and PB1–336 were thus considered to be the host species-associated amino acid positions in influenza A/H1N1 viruses, and that PB1–216G is the human-associated residue, whereas PB1–336I is associated with infections in both swine and humans. At that time, the biological significance of how antigenic variation at PB1 enabled viruses to switch host species was poorly understood. Since then, the pdmH1N1 has become a circulating seasonal human influenza A/H1N1 virus worldwide.

In our current study, we investigated the impact of PB1–216G and PB1–336I on the gene pool of currently circulating seasonal influenza A/H1N1 viruses. We found that the serine-to-glycine point mutation at nucleotide position 216 in PB1 (PB1-S216G) significantly reduced RdRp fidelity. Viruses with PB1–216G demonstrated increased sensitivity to ribavirin and reduced virulence in mice. In cells treated with the NA-specific inhibitor, oseltamivir, PB1–216G viruses generated mutations in NA at a faster rate than PB1–216S viruses. Our findings highlight the need for continuous monitoring to identify emerging adaptive mutations that might contribute to future influenza pandemics.

## Methods

### Viruses and cells

Madin-Darby canine kidney (MDCK; ATCC PTA-6500) cells and human embryonic kidney 293 (HEK293; ATCC CRL-1573) and HEK 293 T (ATCC CRL-3216) cells were grown in complete Dulbecco’s modified Eagle’s medium/high glucose (DMEM/HG) supplemented with 10% fetal bovine serum. All recombinant viruses were generated in vitro by using reverse genetic methods, as previously described [20–22]. The HEK293T cells were transfected using the Polyjet DNA transfection reagent (SignaGen, Rockville, MD, USA). The cells were cotransfected with eight pHW2000-based plasmids (1 mg/plasmid), each of which contained one of the eight genomic segments of influenza A virus. Viral genes were expressed under the control of the dual promoters on pHW2000. The inoculums were removed 6 h post-transfection, and replaced with serum free DMEM/HG medium containing 0.1% trypsin (Life Technologies, Carlsbad, CA, USA). Between 72 and 120 h post-transfection, the culture supernatants were collected for virus recovery. Recombinant viruses were amplified in MDCK cells for 1 to 3 passages before virus titer determination by plaque assay. Single-nucleotide mutations were introduced into the PB1 plasmid by site-directed mutagenesis, as previously described [23]. The full-length sequences of the eight viral genomic segments were obtained by conventional DNA sequencing. Recombinant PR8 virus contains eight viral genomic segment of PR8, PR8^PB1S216G^ virus contains eight viral genomic segment of PR8 but the residue 216 on PB1 is replaced from serine to glycine, and PR8PB1^I563R^ is the residue 563 on PB1 is replaced from Isoleucine to Arginine. Recombinant PR8/TW216^PB1^ virus contains seven viral genomic segment of PR8 and PB1 segment of TW126, and recombinant PR8/TW216^PB1G216S^ virus is only different from recombinant PR8/TW216^PB1^ virus as the residue 216 on PB1 of TW126 is replaced from glycine to serine.

### Virus growth curve assay

MDCK cells were seeded at 5.0 × 10^5^ cells/well in 6-well plates before 24 h infection. MDCK cells were washed with 1 ml PBS twice followed by infecting with viruses at a MOI of 0.001. After incubation 1 h, the cells were washed twice with 1 mL PBS followed by adding 2 ml DMEM/HG medium each well containing 2 mg/ml TPCK-treated trypsin and were incubated at 37 °C. The supernatants were collected at indicated hour post-infection.

### Plaque assay

Viral titer was determined by plaque assay [24]. MDCK cells were seeded at 6.0 × 10^5^ cells/well in 6-well plates before virus infection for 24 h at 37 °C. Virus titers were evaluated by serial 10-fold dilutions in 6-well plates at 37 °C. At 1 h post-infection, cells were washed twice with 1 mL PBS, and the cells overlaid with 2 mL of DMEM/HG medium supplemented with 0.3% agarose. After incubation for 48 h at 37 °C, the cells were fixed in 10% formalin for at least 1 h before crystal violet staining. Virus titers were calculated as the number of plaque forming units (PFU) per milliliter.

### Determination of virulence in mice

All animal experiments were approved by the Institutional Animal Care and Use Committee of National Defense Medical Center (IACUC-10-005). Female BALB/c mice at 4–5 weeks of age were purchased from National Laboratory Animal Center (Taipei, Taiwan), and housed under specific pathogen-free conditions until virus challenge at 6 weeks of age. Four to seven mice per group were anesthetized by intraperitoneal injection of 0.5 mg zolazepam chlorhydrate (Virbac, Carros, France) before intranasal inoculation with 50 μL virus solution containing 200 PFU or serum free DMEM/HG (control). Mice were observed for illness or death for 14 days. Illness was recorded as lethal if mice lost 25% body weight, and euthanization was performed humanely by CO_2_ asphyxiation. For lung titer determination, three to five mice per group were euthanized at 72 h post-infection, and the lungs were homogenized in 1 mL DMEM/HG supplemented with antibiotics and 2.5 μg/mL TPCK-treated trypsin. Homogenates were then centrifuged at 2000×g for 5 min. Supernatants were aliquoted, and stored at − 80 °C for viral titrations. The lung viral titers were determined by plaque assay as described previously [24].

### Quantification of replication capability and mutation frequency of influenza virus by dual luciferase RT2AF reporter system

The HEK293 cells were at 1.0 × 10^5^ cells/well. Cells were transfected with 400 ng RT2AF in 24-well plates using Lipofectamine 2000 (Invitrogen, Carlsbad, CA, USA) in a total volume of 750 μL/well, according to manufacturer’s protocol. Transfection mediums were removed 6 h later, and replaced with fresh medium. At 24 h post-transfection, cells were virus infected (MOI = 0.1). At 48 h post-infection, cells were lysed, and Firefly luciferase (Fluc) and Renilla luciferase (Rluc) activities were measured. Viral replication was measured based on Rluc activity, and the mutation potential was calculated as the cumulative mutation index (CMI), whereby CMI = Fluc/Rluc. For the detail descriptions, please refer to Additional file 1: Figure S2.

### Replication capability and cumulative mutation index (CMI) by dual-luciferase RT2AF reporter

Briefly, influenza virus RdRP minireplicon: 1.0 × 1 0^5^ HEK 293 cells seeded in 24-well plates before 24 h were transfected using Polyjet DNA transfection reagent (SignaGen, Rockville, MD) according to the manufacturer’s protocol (www.signagen.com). Two hundred nanograms of expression plasmids encoding PB2, PA, NP and wild-type PB1 or PB1–216 variant were cotransfected with 200 ng RT2AF reporter (Fig. 3a). After 48 h post transfection, cells were lysed and Firefly (Flu) and Renilla (Rlu) luciferase activities were measured using the Dual-Glo Luciferase Assay System (Promega) according to the manufacturer’s protocol. The replication capability of RdRp by relative Rlu luciferase activity and mutation potential (CMI) by Fluc/Rluc ratio were calculated.

### Ribavirin assay

The HEK293 cells were transfected with RT2AF reporter as described above. At 24 h post-transfection, cells were treated with ribavirin for 4 h, before viral infection. At 48 h post-infection, cells were lysed, and Fluc and Rluc luciferase activities were measured.

### Sequence analysis of influenza A virus

**Fig. 1:**
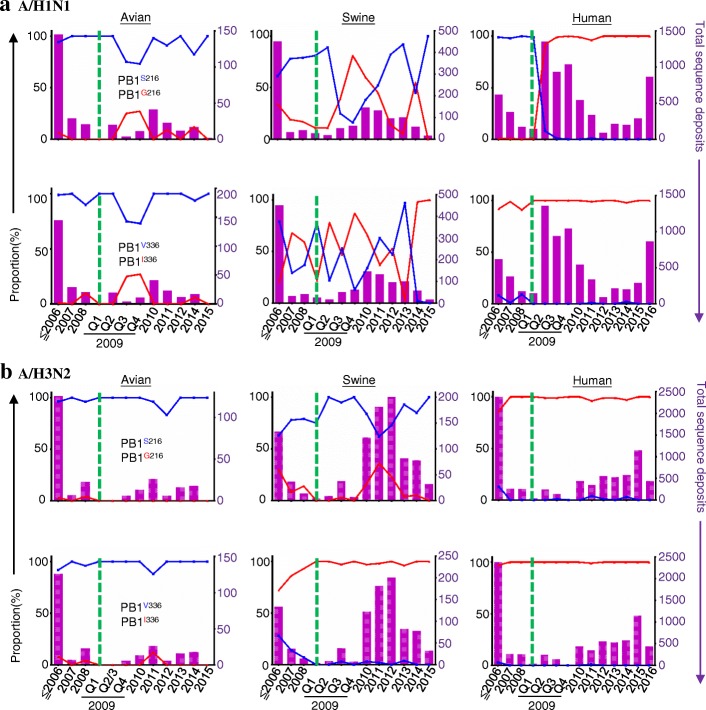
Chronological analysis of PB1–216 and PB1–336 in avian, swine, and human influenza A viruses. Amino acid sequence data of full-length PB1 of for **a** influenza A/H1N1 viruses and **b** influenza A/H3N2 viruses deposited in the Influenza Virus Database (GenBank) prior to August 31, 2016, were analyzed to determine the residue identity at positions 216 and 366 in the PB1 subunit of viral RdRp. The data excluded the 2013 avian A/H1N1 viruses and 2016 avian A/H3N2 viruses because these retrievals contained no full-length PB1 sequences. Blue and red lines represent the percentage of viruses with the residue indicated. Green dotted line denotes the presumed onset of the 2009 influenza A/H1N1 pandemic. Each purple bar represents the numbers of full-length PB1 sequences deposited in year indicated

The PB1 protein sequences of avian, swine, and human H1N1 (Fig. 1a) and H3N2 (Fig. 1b) influenza A viruses were downloaded from the Influenza Virus Resource of GenBank on October 31, 2016. The total number and percentage of each reported amino acid residue was determined for PB1–216 and PB1–336. Genomic sequence (8 genomic segments) data for human A/H1N1 viruses were downloaded from GenBank on March 10, 2018. For the whole viral genome analysis, the various genomes were first divided into two groups based on whether the PB1–216 residue was G or S. The NA segments from each group were then aligned to identify NA mutations in oseltamivir-resistant variants (Table 2).

### Analysis of HA mutation frequency

Wild-type PR8 and PR8^PB1(S216G)^ viruses were passaged twice in MDCK cells at an MOI of 0.001. Viral supernatant for viral RNA was subjected to reverse transcription using the SuperScript III reverse transcriptase (Life Technologies) with universal primer (5′-AGCRAAGCAGG-3′). The HA cDNA was amplified by the Phusion High-Fidelity DNA Polymerase (Thermo Scientific) with forward and reverse primers (5′-AGCAAAAGCAGGGGAAAATA-3′ and 5′-GTCCTGTAACCATCCTCAAT-3′). PCR product was cloned into pJET1.2/blunt using the CloneJET PCR Cloning Kit (Thermo Scientific) according to manufacturer’s protocol. Clones were sequenced in an ABI Prism 3700 sequence analyzer (Applied Biosystems).

### Oseltamivir assay

The HEK293 cells were infected with PR8 or PR8^PB1(S216G)^ (MOI = 0.01) for 48 h. The cells were serial passaged while incrementally increasing the concentration of oseltamivir (Toronto Research Chemicals). The concentrations of oseltamivir from 1 to 625 nM, approximately 0.25- (4.2 nM) to 148-fold IC50 [25], were added gradually up to 625 nM, which were maintained from passage 5 thereafter until passage 14 as described in Additional file 1: Figure S4a. The culture supernatants were collected at 48 h after each passage, as previously described [26].

### Sequencing analysis of *NA*

For the conventional Sanger sequencing analysis, viral RNA purified and reverse transcription described as above. The cDNA was amplified by PCR using NA-specific primer set A (5′-AATGAGCTGCCCTGTCGGTG-3′ and 5′-TACTTGTCAATGSTGAAYGG-3′) or primer set B (5′-AGCAAAAGCAGGAGTTTAAA-3′ and 5′-GGTTTCAGTTATTAGCC GTTG-3′) respectively. The PCR products were subjected to direct Sanger sequencing. For deep sequencing analysis, nucleotides 523–921 of NA, which correspond to amino acids 189–321 in PR8, were sequenced by Genomics Ltd. (Taiwan) on the Illumina MiSeq platform. The cDNA was PCR amplified using the following barcoded primers: PR8 (5′-ACAGTGAATGGGVTGGCTAACAATCGG-3′ and 5′-ACAGTGATGTCACCGAAAA CCCCACTG-3′) and PR8^PB1(S216G)^ (5′-GCCAATAATGGGVTGGC TAACAATCGG-3′ and 5′-GCCAATATGTCACCGAAAACCCCACTG-3′). Total reads obtained were over 2.5 million per strain, and output data were > 2 gb over the 399-bp target with a mean quality score of 38.3 ± 0.8. Output data was first sorted by barcode sequence that represented the NA plasmid, NA of PR8, and NA of PR8^PB1(S216G)^. Given the general error rate of the Invitrogen SuperScript III reverse transcriptase [27] used in this study is 3.4 × 10^− 5^, we arbitrarily defined the cut-off value as > 10 mutations in 1 million reads. Therefore, positions at which mutations occurred at a frequency higher than 10^− 5^ were considered significantly variable.

## Results

### Amino acid position 216 in PB1 of influenza A/H1N1 is a species-associated position that distinguishes between human and avian influenza viruses after the emergence of pdmH1N1

By entropy-based computational approach to characterize avian-human signatures, we have previously identified several human-associated positions on pandemic H1N1 2009 virus genome that were all within the internal genes of RdRP complex [19]. In fact, this pandemic 2009 virus strain had subsequently become a human seasonal influenza A/H1N1 strain currently circulating worldwide. We were interested in monitoring the characteristic change in amino acids that may be attributed to the emergence of pandemic 2009 virus and its current offspring of human seasonal A/H1N1. From our previous study, PB1–216 was one of the species-associated positions identified for its exclusively human-like residue Gly found in all pandemic 2009 H1N1 viruses and yet, before this pandemic episode, most human influenza A/H1N1 deposited were an avian signature Ser at PB1–216 as their coexisting avian A/H1N1 influenza viruses [19]. In contrast, regardless of pandemic impact, we observed PB1–336 of pandemic H1N1 2009 virus still remained human-like residue Ile while avian influenza H1N1 were almost an avian-associated Val [19]. This observation implies there may be a major transitioning pattern from avian to human occurred amongst different influenza A virus populations during pandemic 2009 outbreak. To further understand the significance of the point mutations PB1-S216G and PB1-V336I in pdmH1N1, we first investigated the chronological changes at these nucleotide positions between A/H1N1 and A/H3N2 viruses collected from different hosts of avian, swine, and human deposited in the Influenza Virus Database. We found that most of the avian A/H1N1 viruses contained PB1–216S and PB1–336 V both before and after 2009 (Fig. 1a). Most of the human A/H1N1 viruses also contained the avian-associated residue, serine, at PB1–216 before 2009. However, with the emergence of pdmH1N1 in 2009, most of the human A/H1N1 viruses primarily had PB1–216G likely via genome reassortment, which thereafter remained the most prevalent residue glycine at that position. By contrast, most human A/H1N1 viruses contained the mammalian-associated residue, isoleucine, at PB1–336 both before and after 2009. Swine A/H1N1 viruses exhibited frequent substitutions between the avian- and mammalian-associated residues at both PB1–216 and PB1–336, reflecting the susceptibility of swine to both avian and human influenza viruses and humans on the other hand can be also infected by swine influenza viruses.

In A/H3N2 viruses, some substitutions between avian and mammalian signatures were readily observed at PB1–216 and PB1–336 collected from avian and swine viruses (Fig. 1b), with essentially none of the human A/H3N2 viruses exhibiting such changes before or after the emergence of pdmH1N1. Indeed, PB1–216G was most prevalent among human A/H3N2 viruses long before 2009. Given that the PB1 genomic segment of pdmH1N1 virus was recently acquired from a human seasonal A/H3N2 virus [3], we examined sequences deposited before 2006 to determine whether an avian to mammalian substitution had previously occurred in human A/H3N2 viruses. We found that the PB1-S216G point mutation indeed occurred in 1993, after which PB1–216G remained the most prevalent signature in PB1 in human A/H3N2 viruses (Additional file 1: Figure S1). These observations suggested that the substitution from the avian-associated residue, serine, to the human-associated residue, glycine, at PB1–216 at the onset of the 2009 pandemic was the result of robust evolutionary adaptation that has impacted currently circulating seasonal human A/H1N1 viruses worldwide.

### PB1-S216G in influenza A/H1N1 viruses attenuates virulence in mice

The A/Taiwan/126/2009 (TW126) virus, a clinical isolate of pdmH1N1 from Taiwan [24] that contained PB1–216G, was reported to be less virulent in mice, as compared with the A/Puerto Rico/8/1934 (PR8) virus [28], a widely-used influenza A/H1N1 laboratory reference strain containing PB1–216S [19]. TW126 PB1 contains the typical human signatures of Gly at PB1–216 and Ile at PB1–336; in contrast, the laboratory reference strain, PR8, exhibits avian signatures at the corresponding positions of PB1. To examine whether this difference in pathogenicity in mice was associated with the avian and mammalian signatures in the PB1 sequences of TW126 and PR8, we first used a reverse genetic to recover PR8/TW126^PB1^, a reassortant PR8 virus containing the whole PB1 genomic segment of TW126. We also generated the PR8/TW126^PB1(G216S)^ variant by PB1-G216S point mutation in PR8/TW126^PB1^. To evaluate the effects of these mutations, we compared the replication and virulence of PR8/TW126^PB1^, PR8/TW126^PB1(G216S)^, and parental PR8 in Madin-Darby canine kidney (MDCK) cells and intranasally inoculated BALB/c mice.

**Fig. 2:**
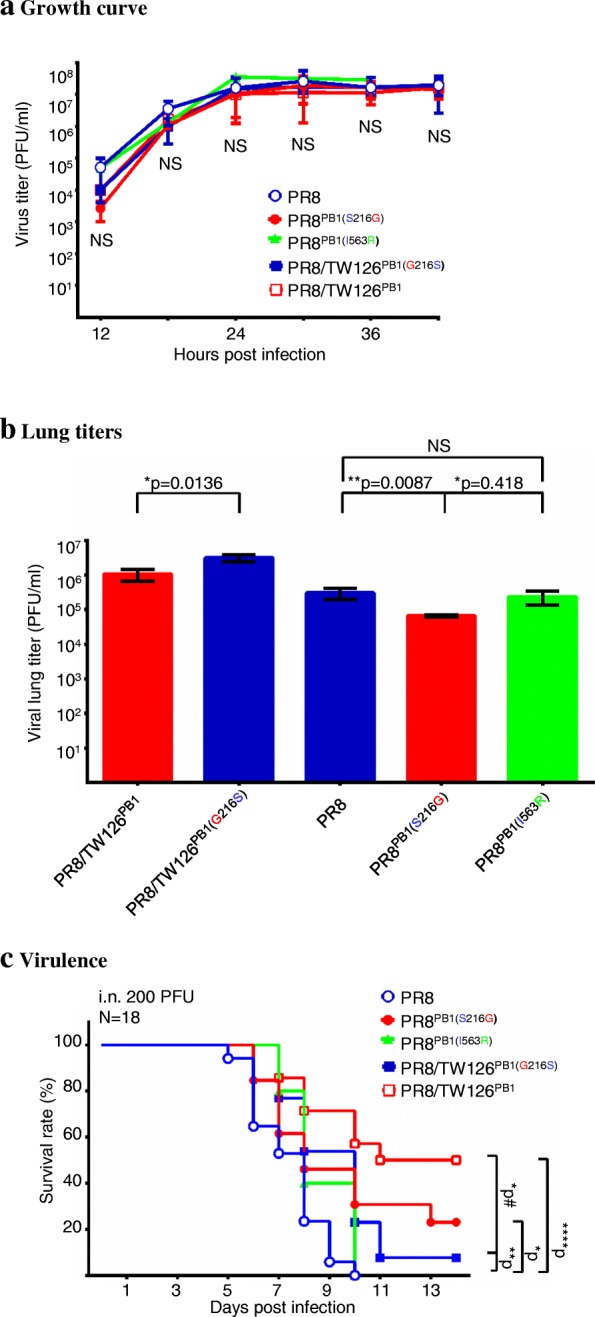
Effects of the PB1-S216G mutation on virus replication and virulence in mice. **a** Growth curves for the PR8, PR8^PB1(S216G)^, PR8^PB1(I563R)^, PR8/TW126^PB1(G216S)^, and PR8/TW126^PB1^ viruses in MDCK cells at 12–42 h post-infection. **b** Virus titers in lung tissue homogenates from 18 female BALB/c mice infected PR8, PR8^PB1(S216G)^, PR8/TW126^PB1(G216S)^, and PR8/TW126^PB1^ viruses at 72 h post-infection were determined by plaque assay. Error bars, standard error of the mean of three independent experiments; NS, not significant (*p* > 0.05) by Student’s t-test for (**a**-**b**). **c** Groups of 18 female BALB/c mice at 6 weeks of age were challenged by infections of PR8, PR8/TW126^PB1^, PR8/TW126^PB1(G216S)^, PR8^PB1(S216G)^, or PR8^PB1(I563R)^. Survival rates of the infected mice were recorded daily for 14 days. Log-rank (Mantel–Cox) test was used to confirm the statistically significant differences in survival rate. *#*p* = 0.032; *p = 0.0258; **p = 0.0048; and ****p < 0.0001

Growth curves for PR8/TW126^PB1^, PR8/TW126^PB1(G216S)^, and PR8 in MDCK cells were similar (*p* > 0.05; Fig. 2a). In mice, infection with PR8/TW126^PB1(G216S)^ caused significantly higher lung-tissue virus titers (*p* = 0.0136) and lower survival (*p* = 0.032), as compared with PR8/TW126^PB1^ (Fig. 2b, c) that exhibited significantly higher survival than both PR8 (*p* < 0.0001) and PR8/TW126^PB1(G216S)^ (*p* = 0.032). To further investigate the role of PB1–216S in virulence in mice, we used PR8 virus to generate the PR8^PB1(S216G)^ and PR8^PB1(I563R)^ variants. We used the PR8^PB1(I563R)^ variant as a control for our experiments because the I563R mutation in PB1 was not expected to affect polymerase activity or fidelity. Growth curves of PR8^PB1(S216G)^ in MDCK cells were similar to those of PR8, PR8/TW126^PB1^, PR8/TW126^PB1(G216S)^, and PR8^PB1(I563R)^ (*p* > 0.05 for all; Fig. 2a). In mice infected with PR8^PB1(I563R)^, lung-tissue virus titers and survival were similar to those for PR8-infected mice (*p* > 0.05 for both; Fig. 2b, c). Mice infected with PR8^PB1(S216G)^ had significantly lower lung-tissue virus titers (*p* = 0.0087), and exhibited improved survival (*p* = 0.0258), as compared with PR8-infected mice. These results indicated that avian-associated PB1–216S is a major virulence determinant for influenza A/H1N1 viruses in mice. By the lethal challenge test in mice, the biological significance could be readily observed because of the difference between PB1–216S and PB1–216G containing viruses (Fig. 2c); influenza A viruses with PB1–216S appeared to be more virulent than virus with PB1–216G.

### PB1-S216G in A/H1N1 viruses contributes to higher mutation frequency at comparable replication levels

**Fig. 3:**
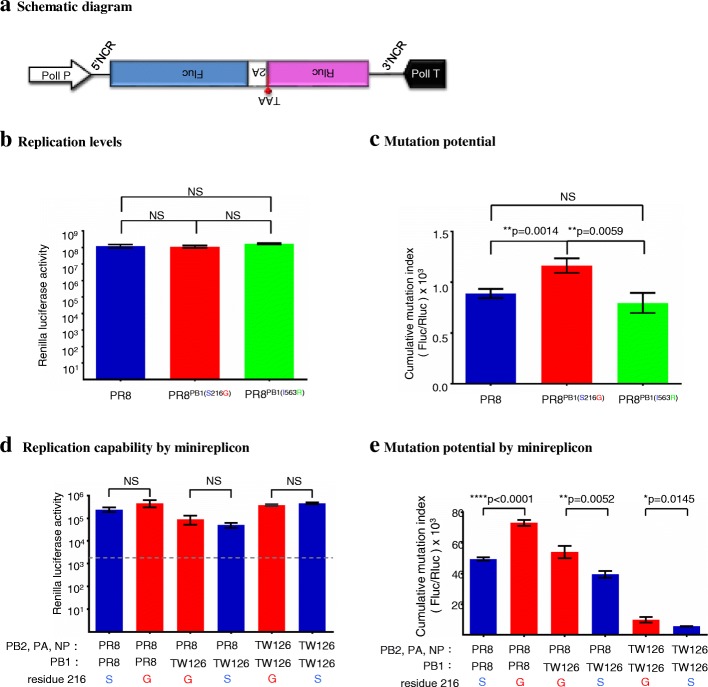
Effects of the PB1-S216G mutation on replication capability and mutation potential in virus-infected cells using the dual-luciferase RT2AF reporter. **a** Schematic diagram of mutability assay for influenza RdRp. The dual-luciferase RT2AF reporter is flanked by the 5′ and 3′ UTR sequences of the WSN-NP genome, and transcription was controlled by the human *PolI* promoter and the murine terminator. **b** Replication capability was calculated based on Rluc luciferase activity and **c** the mutation potential was calculated as cumulative mutation index (CMI) based on the Fluc/Rluc ratio. **d** and **e** Mutation potential of RdRp from PB1–216 variants was measured by influenza minireplicon system. The PB2, PA, NP expression plasmids plus wild-type PB1 or PB1–216 variant plasmids were co-transfected with RT2AF reporter in HEK 293 cells. After 72 h, the replication capability (**d**) by Rlu luciferase activity and mutation potential (Cumulative Mutation Index; CMI) (**e**) by Fluc/Rluc ratio were evaluated in the indicated PB1 plasmids containing either 216S or 216G, respectively. Error bars indicate the standard error of the mean of three independent experiments. Student’s two-tailed unpaired t-test was performed to determine the *P* value; NS, not significant (*p* > 0.05)

Viral RNA reporter genes have been used to quantify the replication and mutation frequency of HIV reverse transcriptase [29] and the RdRp of Cucumber mosaic virus [30]. Because RdRp fidelity has been associated with the virulence of influenza A viruses in mice [31], we investigated whether the PB1-S216G point mutation affects mutation frequency of PR8 and PR8^PB1(S216G)^. We constructed an artificial influenza genomic segment containing the dual luciferase RNA reporter gene [32], RT2AF, which contained the open reading frames for Firefly and Renilla luciferases connected in tandem by a UAA stop codon (Fig. 3a). Replication capability was determined based on Renilla luciferase (Rluc) activity. The ratio of the activities of Firefly luciferase (Fluc) and Rluc was used to quantify RdRp fidelity based on the frequency of stop codon repair during viral replication and transcription of RT2AF, which was expressed as the cumulative mutation index (CMI), whereby CMI = Fluc/Rluc (Additional file 1: Figure S2). In influenza-virus-infected HEK293 cells transfected with RT2AF, we found that, while virus replication levels were similar between PR8, PR8^PB1(S216G)^ and PR8^PB1(I563R)^ (Fig. 3b), the PR8^PB1(S216G)^ variant exhibited a significantly higher CMI as compared with PR8 and PR8^PB1(I563R)^ (*p* = 0.0014 and *p* = 0.0059, respectively; Fig. 3c).

In addition, combined with expression constructs for the polymerase subunits PB2, PB1, PA and NP proteins, this dual-luciferase RT2AF reporter replicon system allows to quickly measure replication capability and evaluate mutation potential for the given influenza RdRp activity using various PB1–216 variants. Using such minireplicon assay system, we in Fig. 3d and e compared the replication capability and mutation potential between pairs of PR8 (PB2 + PA + NP)/PR8 (PB1–216S) and PR8 (PB2 + PA + NP)/PR8 (PB1-S216G), PR8 (PB2 + PA + NP)/TW126 (PB1–216G) and PR8 (PB2 + PA + NP)/TW126 (PB1-G216S), as well as TW126 (PB2 + PA + NP)/TW126 (PB1–216G) and TW126 (PB2 + PA + NP)/TW126 (PB1-G216S), respectively. The replication capability of RdRp by measuring Rluc activity were not significantly different (Fig. 3d), indicating that swapping between Gly and Ser at PB1–216 did not affect RdRp replication levels. However, the mutation potential of RdRp determined by CMI (Fig. 3e) showed that RdRp complex having PB1–216G derived from different viruses could all exhibit significantly higher mutation capability than PB1–216S; especially for the experimental groups of TW126 (PB2 + PA + NP)/TW126 (PB1–216G) and TW126 (PB2 + PA + NP)/TW126 (PB1-G216S), from which all four polymerase subunits of PB2, PB1, PA and NP proteins could be closely interactive during viral replication as they were in the native background of TW126 virus, a clinical isolate of pdmH1N1 from Taiwan. In this study, the results from Fig. 3 were further confirmed by using a previously described conventional assay for determining RdRp nucleotide incorporation fidelity and clonal sequencing [31] that had compared the frequency of mutations in HA of PR8 with that of PR8^PB1(S216G)^. As these results shown in Table 1, the mutation frequency of PR8^PB1(S216G)^ was significantly greater than that of PR8 (*p* = 0.025). The combined results of our experiments indicated that the PB1–216G mammalian signature in influenza A/H1N1 viruses confers lower RdRp fidelity than the PB1–216S avian signature, and thereby increases the frequency of mutations during viral replication.

**Table 1:**
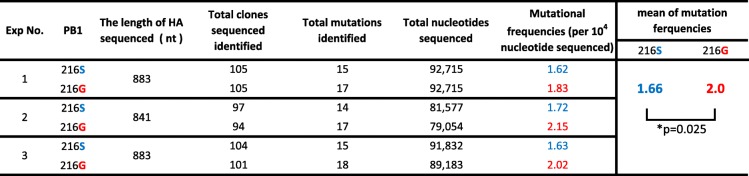
Mutation frequency of influenza A/H1N1 viruses based on conventional sequencing analysis

Wild-type PR8 and PR8^PB1(S216G)^ viruses were passaged twice in MDCK cells. The *HA* cDNA was amplified from viral supernatant, and cloned into pJET1.2/blunt as described in the Methods. The *HA* mutation frequency was determined by clonal sequencing of 94 to 105 bacterial clones for each experiment were picked and repeated independently three times. *by Student’s two-tailed unpaired *t*-test; *NS* Not significant

### PB1-S216G in influenza A/H1N1 viruses increases sensitivity to ribavirin

**Fig. 4:**
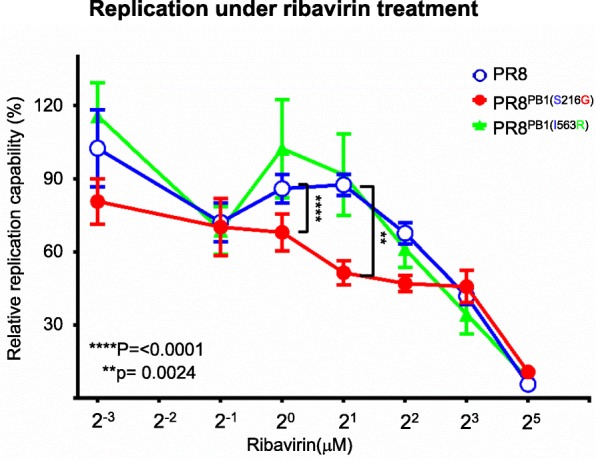
Effects of the PB1-S216G mutation on influenza A/H1N1 virus replication capability and adaptability in cells treated with ribavirin. The replication capability of the PR8 and PR8^PB1(S216G)^ viruses was measured in the presence of ribavirin. HEK293 cells were transfected with RT2AF-transfected for 4 h, and the cells were infected with PR8, PR8^PB1(S216G)^, or PR8^PB1(I563R)^ virus in the presence of the indicated concentrations of ribavirin. At 48 h post-infection, cell lysates were prepared, and the Rluc and Flu luciferase activities were measured. The relative replication capability was determined by Rluc/Rluc (without ribavirin) ratio. Error bars, standard error of the mean of three independent experiments; NS, not significant (*p* > 0.05) by Student’s two-tailed unpaired *t*-test; ****p < 0.0001; and **p = 0.0024

Ribavirin is a nucleotide analog targeting RdRp that causes lethal hypermutation in RNA viruses [28, 33]. In a previous study a mutant variant of poliovirus that exhibited enhanced RdRp fidelity was more resistant to ribavirin treatment than wild-type poliovirus with low RdRp fidelity [12], likely because such a faithful RdRp generated less mutations during viral replication. Similar observations were also reported for HIV studies [34, 35], in which the high fidelity of reverse transcriptase could reduce the lethal toxicity of nucleoside analogue 2′,3′-dideoxy-3′-thiacytidine (3TC) by making less mutations during retroviral replication. In this study, to examine the effect of the PB1-S216G point mutation on resistance to ribavirin treatment, we compared the replication of PR8 and PR8^PB1(S216G)^ viruses in RT2AF-transfected HEK293 cells in the presence of various concentrations of ribavirin. We found that the ribavirin-mediated inhibition of PR8^PB1(S216G)^ replication was significantly greater than that of PR8 especially at 1 and 2 μM ribavirin (Fig. 4). This result indicated that the PB1-S216G point mutation of influenza A virus could not only reduce RdRp fidelity but also increase sensitivity to ribavirin during replication, consistent with the results from the previous reports concerning poliovirus and HIV [12, 34, 35].

### PB1-S216G in A/H1N1 viruses increases NA mutation potential under oseltamivir selection

There has been considerable discussion concerning how low replication fidelity in RNA viruses might contribute to adaptive evolution and enhanced viral fitness in infected hosts by increasing genetic diversity in virus populations [27]. A previous study of chikungunya virus variants found that increased RdRp fidelity resulted in reduced genetic diversity and lower fitness in natural mosquito hosts and newborn mice, compared with that of a wild-type chikungunya virus [15]. In a previous study of poliovirus variants, increased fidelity resulted in a less diversified population and reduced adaptation under adverse growth conditions [11, 12]. The V43I mutation in influenza PB1 is associated with high-fidelity RdRp [31]. Our analysis of influenza A virus sequences showed that PB1–43I did not appear in human influenza A viruses, and occurred only rarely in avian H5N1, swine H3N2, and swine H1N1 viruses (Additional file 1: Figure S3), thereby excluding it as a factor in the displacement of PB1–216S viruses by PB1–216G viruses.

Viral neuraminidase inhibitors (NAIs), such as oseltamivir, are not mutagens per se, but do contribute to the emergence of NAI-resistant mutations within the NA gene as the result of random RdRp-mediated nucleotide misincorporation during viral replication in NAI-treated cells. Previous studies of influenza A/H1N1viruses have reported NA mutations, including NA-H274Y/H275Y and NA-N294S, that contributed to oseltamivir resistance [26, 36–38]. To gain insight into how low-fidelity PB1–216G variants replaced PB1–216S after the emergence of pdmH1N1, we examined the incidence of mutations that conferred oseltamivir resistance from 2006 to 2017. We found that the incidence of oseltamivir resistance in 2006 and 2007 was much lower than that in 2008, after which an NA-H275Y point mutation coincided with a steep rise in the incidence of oseltamivir resistant mutations worldwide (Additional file 1: Table S1). In mid-2009, the oseltamivir-sensitive pdmH1N1 virus (PB1–216G/NA-275H) emerged (Fig. 1a), the number of oseltamivir-resistant NA-275Y viruses decreased rapidly. Thus, the rapid displacement of NA-275Y by NA-275H coincided with the displacement of the avian 216S signature by the mammalian 216G signature in the field.

The data in Additional file 1: Table S1 also show that five distinctly different NA mutations conferring oseltamivir resistance (S246 N, D198G, D198N, D198GY, and Y155H) occurred after the 2009 pandemic. This pattern in oseltamivir resistance was confirmed in the whole genome analysis, which showed that most NA-275H/PB1–216G viruses were oseltamivir sensitive, and oseltamivir resistant NA-275Y/PB1–216S viruses contained at least one of these five NA mutations (Table 2). We then examined the incidence of the permissive secondary mutations, NA-V241I and NA-N369K, which have been shown to that improve fitness in NA-275Y viruses [39]. The incidence of NA-241I and NA-369 K decreased during the first half of 2009; however, the number of viruses with NA-241I or NA-369Ks increased after 2009, and became predominant by 2011 (Additional file 1: Table S1).

**Table 2:**
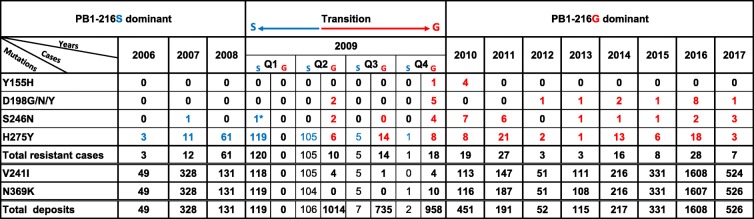
Whole genome analysis of correlation between PB1-S216G and NA mutations conferring oseltamivir resistance in human influenza A/H1N1 viruses

Genome sets were retrieved from the Influenza Virus Database (GenBank). The *NA* sequences for PB1-216S viruses (blue) and PB1–216G viruses (red) were aligned and analyzed to identify NA mutations conferring oseltamivir resistance and permissive secondary mutations in NA (V241I and N369K)

To investigate whether RdRp fidelity affects influenza A virus adaptation under a stress, we used a modified version of a previously described method for the in vitro selection of oseltamivir-resistant pdmH1N1 variants [26] to assess the effects of the PB1-S216G point mutation on the frequency of mutations in NA conferring oseltamivir resistance in PR8. As shown in Additional file 1: Figure S4a, PR8 and PR8^PB1(S216G)^ infected MDCK cells were serial passaged with successive incubation in progressively higher concentrations of oseltamivir, reaching a maximum concentration of 625 nM oseltamivir at passage 5 and thereafter. The NA gene sequence was determined after each passage by conventional Sanger sequencing of viral cDNA, which allowed the identification of mutations in a single, relatively long read without the assembly and annotation of shotgun sequencing data obtained using high-throughput methods [36]. The NA^N294S^ mutation was first detected in PR8^PB1(S216G)^ at passage 7, and serine was the most prevalent residue at NA-294 at passage 9 and thereafter (Additional file 1: Figure S4b, right). No other mutations were detected in the NA gene of PR8^PB1(S216G)^. The NA^H274Y^ mutation was first detected at passage 11 in PR8 that had PB1–216S, and tyrosine became the most prevalent residue at NA-274 at passage 14 (Additional file 1: Figure S4b, left). These results illustrate that PB1-S216G in A/H1N1 viruses could acquire adaptive mutations in NA conferring oseltamivir resistance at a higher frequency than viruses with PB1-G216S in A/H1N1 viruses. Nevertheless, it remains unclear as how two different NA mutations could have independently emerged from PR8 or PR8^PB1(S216G)^ under selection pressure.

Given that the virus strains used in the above-mentioned experiments were synchronized at passage 5, we investigated whether NA^N294S^ and NA^H274Y^ were merely pre-existing mutants in the PR8^PB1(S216G)^ and PR8 populations, respectively. Following reverse genetics recovery, nucleotides 523 to 921 (amino acid positions 189 to 321) in the *NA* gene of PR8 and PR8^PB1(S216G)^ at passage 5 were subjected to deep sequencing. This region was selected because it included all the positions at which we had previously identified NA mutations conferring oseltamivir resistance by Sanger sequencing, and previous studies have shown that the majority of mutations conferring NAI resistance in influenza A viruses have occurred in this region of the *NA* gene [40].

**Fig. 5:**
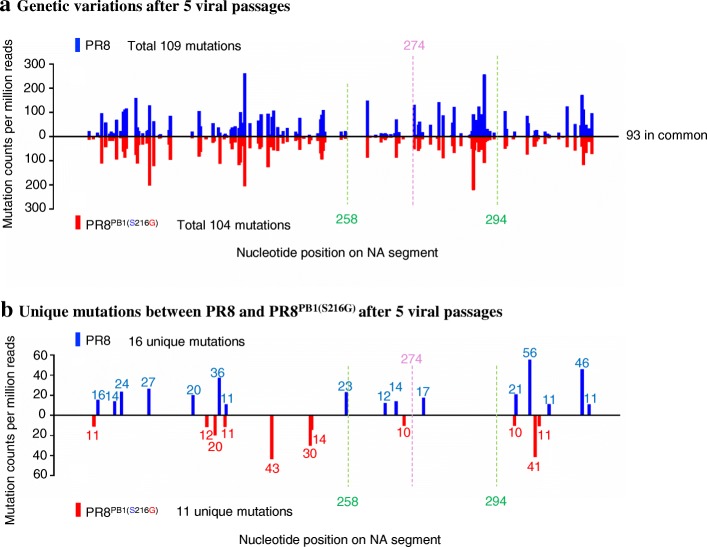
Genetic landscape of NA in PR8 and PR8^PB1(S216G)^ viruses. **a** After being reverse-genetically recovered, PR8 and PR8^PB1(S216G)^ viruses were synchronized at passage 5, and successively amplified during nine additional passages. Nucleotides 523–921 (amino acids 189–321) were deep-sequenced, and the NA mutations were plotted as mutation counts per million reads versus nucleotide position. Both viruses appeared to establish their own unique genetic landscapes after five serial passages, and yet no NA mutations associated with oseltamivir resistance were detected from either virus. **b** Identification of unique NA mutations in PR8 and PR8^PB1(S216G)^ at passage 5, as compared to their parental viruses at passage 1. In PR8, 16 unique mutations were identified at passage 5, whereas 11 unique mutations were detected in PR8^PB1(S216G)^ at passage 5

Our analysis showed that, at passage 5, a total of 104 and 109 mutations had occurred in PR8^PB1(S216G)^ and PR8, respectively, among which 93 were shared between these two viruses (Fig. 5a). The remaining mutations in PR8^PB1(S216G)^ (*n* = 11) and PR8 (*n* = 16) were unique to each virus (Fig. 5b). In addition, at passage 5 there were no oseltamivir-resistant mutations of NA^N294S^ and NA^H274Y^ could be detected in either PR8^PB1(S216G)^ or PR8 by NGS analysis, which strongly suggests that these were adaptive mutations that conferred NAI resistance in cell-based selection system observed in Additional file 1: Figure S4a and b. Moreover, these results showed that both PR8 and PR8^PB1(S216G)^ generated comparable levels of population diversity in the absence of substantial selective pressure, as evidenced by the similarly large number of mutations that were present in each virus at passage 5 (Fig. 5b).

## Discussion

Although most human A/H3N2 viruses had avian-associated PB1–216S from 1968 to 1991, a substitution to mammalian-associated PB1–216G occurred near the end of that period, and PB1–216G became predominant in human A/H3N2 viruses thereafter (Additional file 1: Figure S1). In 2009, an A/H1N1 virus contained this mammalian PB1–216G signature by PB1 reassortment originated from A/H3N2, resulting in the emergence of pdmH1N1 (Fig. 1a). We in this study investigated the biological significance of the PB1-S216G point mutation in human A/H1N1 viruses.

The virulence of pdmH1N1 in mice has been shown to be less than that of PR8, an A/H1N1 reference strain containing the avian signature, PB1–216S [28]. We found that PR8 was more virulent in mice than the reassortant virus, PR8/TW126^PB1^, which contains the mammalian PB1–216G signature, despite sharing 99% homology with PR8 PB1 (Fig. 2c). Producing the avian signature at PB1–216 in PR8/TW126^PB1(G216S)^ virus appeared to restore virulence to a level similar to that of PR8 (Fig. 2c), and producing the mammalian signature in PR8^PB1(S216G)^ virus on the other hand reduced virulence, as compared to PR8 (Fig. 2c). These results clearly demonstrated the importance of PB1–216 as a virulence determinant for influenza A/H1N1 viruses in mice.

Using ribavirin to select for resistant viruses, the molecular basis of fidelity determinants within the RdRp gene have been identified for some RNA viruses, including poliovirus [12, 13], Chikungunya virus [15], and the influenza A/H3N2 and H5N1 viruses [31]. Ribavirin-resistant viruses typically contained mutations within the RdRp gene causing elevated-fidelity phenotypes that exhibited lower fitness and/or lower virulence in infected animals, compared with the parental viruses. Among these high-fidelity RdRp mutations in ribavirin-resistant variants, the PB1^V43I^ variant of influenza A/H5N1 exhibited reduced virus population diversity, attenuated virulence, and low neurotropism in mice [31]. By contrast, the mutation of key residues in the RdRp of Coxsackievirus B3 [16] and the exoribonuclease of coronaviruses [17, 18] generated virus variants exhibiting elevated mutation frequencies and attenuated virulence in mice.

We have previously identified several host-specific amino-acid signatures that separated avian and swine viruses from human influenza viruses via entropy-based algorithm analysis of influenza A/H1N1 sequences deposited in GenBank [19, 41]. The presence of these host-specific signatures in human influenza A/H1N1 viruses suggested that these mutations were the result of adaptive genetic evolution that enabled these influenza viruses to circumvent host barriers, which likely resulted in cross-species transmission. Our data show that the avian-to-mammalian signature substitution (serine-to-glycine) at PB1–216 in pdmH1N1 is highly suggestive of avian/swine to human influenza virus transmission contributing to the influenza outbreak that caused the 2009 pandemic [19, 42]. However, the mechanisms through which this host-signature substitution ultimately affected the virulence and fitness of pdmH1N1 has remained unclear. It is worth further investigating how the change of 3-D structure of viral polymerase complex at PB1–216 between serine and glycine in influenza A/H1N1 fine-tunes RdRp’s fidelity during virus replication.

The results of our current study further showed that this natural switch from serine to glycine at PB1–216 increased the mutation frequency of pdmH1N1 by reducing the fidelity of RdRp (Fig. 3; Table 1). The A/H1N1 viruses with PB1–216G were more sensitive to ribavirin inhibition (Fig. 4), and acquired oseltamivir-resistant mutations in vitro at a faster rate than those with PB1–216S in cell-based selection system. Although the mutation potential of PR8^PB1(S216G)^ was greater than that of PR8 as the result of reduced RdRp fidelity in the PB1–216G variant, similar levels of genetic diversity were observed in the population of each virus (Fig. 5), an observation that contrasts sharply with the reduced genetic diversity reported for viruses with high-fidelity RdRp mutations [31]. In addition, the virulence of the low-fidelity PR8^PB1(S216G)^ virus in mice was attenuated relative to that of the PR8 parent virus (Fig. 2c), which is inconsistent with the attenuated phenotype previously reported for a high-fidelity influenza A/H5N1^PB1(V43I)^ variant obtained under ribavirin selection [31] However, this observation was consistent with the previously reports concerning certain viruses with lowered fidelity indeed displayed an attenuated property in vivo [16–18].

We determined that the difference in mutation frequency between PR8 and PR8^PB1(S216G)^ was approximately 20% by conventional sequencing (Table 1) and approximately 30% by minireplicon reporter assay (Fig. 3). Although these differences in mutation frequency are relatively small, the effects of the PB1-S216G point mutation in PR8^PB1(S216G)^ on replication and adaptation, compared to that of PR8, were readily apparent in the results of the ribavirin inhibition assay (Fig. 4) and in vitro NAI assay (Additional file 1: Figure S4). These observations indicate that influenza viruses that may differ subtly in RdRp fidelity can generate a variety of variants under clinically relevant conditions, exhibit similar infectivity and growth characteristics, and generate comparable levels of population diversity.

Since the 2009 pandemic outbreak, human influenza A/H1N1 viruses with PB1–216G have become widespread worldwide, and are now major seasonal influenza viruses that currently co-circulate with influenza A/H3N2 and influenza B viruses. In contrast to the current exclusively predominance of PB1–216G in human A/H1N1 viruses, our analysis of influenza A virus sequences deposited in GenBank (Fig. 1; Additional file 1: Figure S3) showed that PB1–216S remained predominant in avian influenza A/H1N1 viruses. We also found that, while the PB1–43I mutation associated with high-fidelity RdRp [31] occurred rarely in avian H5N1 and swine H3N2 as well as swine H1N1 viruses, it was not found in human influenza A viruses. These results indicated that, although the high-fidelity PB1–43I influenza A variant can be obtained by reverse genetics and occurs sporadically in the field, it confers no significant evolutionary advantage relative to PB1–43 V viruses.

We focused our investigation on identifying the biological mechanism by which the low-fidelity PB1–216G variant replaced the human influenza A/H1N1 viruses with PB1–216S that existed prior to the 2009 influenza pandemic. We found that the incidences of oseltamivir resistance in human influenza A/H1N1 viruses in 2006 and 2007 were much lower than that in 2008, at which time the emergence of oseltamivir resistant variants rose steeply worldwide due to an NA-H275Y point mutation. The frequency of NA-275Y peaked in the first quarter of 2009, with nearly all NA deposits containing the mutation (Additional file 1: Table S1). However, during the mid-2009, the oseltamivir-sensitive pdmH1N1 virus emerged, which contained NA-275H, and pdmH1N1 rapidly displaced oseltamivir-resistant NA-275Y viruses afterwards. The proportion of oseltamivir-resistant NA-275Y A/H1N1 viruses with avian-associated PB1–216S increased rapidly to predominance in the human influenza A/H1N1 population in the first half of 2009, and were subsequently replaced with oseltamivir-sensitive pdmH1N1 at an equally rapid rate (Additional file 1: Table S1). Nonetheless, this change in oseltamivir sensitivity at the population level coincided closely with the rising prevalence of mammalian-associated low-fidelity PB1–216G in influenza A/H1N1 viruses near the onset of the 2009 influenza pandemic (Fig. 1; Table 2). One appealing hypothesis is that the higher-fidelity RdRp (PB1–216S) of the oseltamivir-resistant NA-275Y A/H1N1 viruses resulted in higher replication rate in the absence oseltamivir selective pressure, thereby rapidly increasing the proportion of these viruses in the population during the swine influenza pandemic in the first quarter of 2009. This rapid rise to predominance was followed by the development of a certain unidentified environmental bottleneck(s) plus the acquisition of low-fidelity PB1–216G by an NA-275H A/H1N1 virus that remained continuously present in the population, which allowed it to undergo adaptive mutation at a higher rate than that of the higher-fidelity oseltamivir-resistant NA-275Y/PB1–216S viruses, thereby contributing to the extinction of PB1–216S viruses following the 2009 pandemic.

The data in Additional file 1: Table S1 show that, prior to the midpoint of 2009, oseltamivir resistance was associated almost exclusively with NA-275Y viruses, whereas five additional, distinctly different NA mutations (S246 N, D198G, D198N, D198GY, and Y155H) were also associated with oseltamivir resistance following the 2009 pandemic. Whole genome analysis focusing on the *PB1* and *NA* sequences confirmed this pattern in oseltamivir resistance in NA-H275Y/PB1–216S viruses, and showed that, while most NA-H275H/PB1–216G viruses were oseltamivir sensitive, those that were resistant to oseltamivir contained at least one of the above-mentioned NA mutations, in addition to the predominant H275Y (Table 2). The lower prevalence of oseltamivir-resistant NA-H275Y in human PB1–216G-A/H1N1 viruses was apparently not due to the absence of the permissive secondary mutations, NA-241I and NA-369 K, that confer robust fitness in NA-275Y viruses [39] Although the prevalence of NA-241I and NA-369 K decreased temporarily during the second half of 2009, both of these permissive secondary mutations had become predominant once again in 2011 (Additional file 1: Table S1). These observations suggested that the greater diversity of NA mutations conferring oseltamivir resistance among current human A/H1N1 influenza viruses is the result of higher mutation frequency due to PB1–216G-driven low-fidelity RdRp, which facilitates adaptive mutations in NA under the selective pressure of NAIs, such as oseltamivir.

## Conclusions

In summary, we found that naturally occurring mutations at PB1–216 in influenza A/H1N1 viruses affect replication fidelity, virulence, and adaptability. Our results suggest that the presence of the mammalian signature, PB1–216G, in human A/H1N1 viruses reduces RdRp fidelity, which confers a growth advantage by increasing the probability of adaptive mutations, relative to that of human A/H1N1 viruses bearing the avian signature, PB1–216S. Our results also suggest that, with a canonical low-fidelity RdRp, the increased adaptability and fitness of PB1–216G viruses in human host were the biological mechanisms underlying the replacement of PB1–216S viruses with relative higher fidelity RdRp following the emergence of pdmH1N1. Therefore, continued surveillance of such naturally occurring PB1–216 variants among others is warranted to assess the potential impact of changes in RdRp fidelity on the adaptability and epidemiological fitness of human A/H1N1 influenza viruses.

## Additional file

Additional file 1 : **Table S1** Epidemiological study on oseltamivir-resistant mutations in NA gene of human influenza A/H1N1 viruses using sequences deposited in NCBI influenza database. **Figure S1** Evolution analysis of PB1–216 in influenza A/H3N2 shows serine-to-glycine point mutation at PB1–216 occurred in 1993. **Figure S2** Schematic Diagram of influenza artificial genome containing dual-luciferase RT2AF for measuring RdRp fidelity during influenza virus replication. In the influenza virus-infected and RT2AF-transfected HEK cells, PolI starts to transcribe RT2AF as negative-strand viral RNA, which initiates self-replication of RT2AF reporter. The purpose of this reporter is to not only normalize total replication capability with the first Rluc activities but also evaluate the mutational potential that result in expression of the downstream Fluc activities. The Rluc activity reflects influenza replication levels. The Fluc activity measures the events in which RdRp repaired the engineered stop codon between the Rluc and Fluc reporters. The replication-driven Fluc activity thus represents the mutation potential of the virus. We calculated CMI based on the ratio of Fluc/Rluc that serves as an arbitrary measure of the number of mutation events occurring during virus replication and/or viral transcription. **Figure S3** Epidemiological survey of residue substitution at PB1–43 and PB1–216 of influenza A viruses. Residues at PB1–43 of A/H5N1, H3N2 and H1N1 viruses and residues at PB1–216 of A/H1N1 viruses were examined using data derived from the Influenza Virus Database (GenBank) deposited prior to October 31, 2017. **Figure S4** Effects of the PB1-S216G mutation on influenza A/H1N1 virus replication capability and adaptability in cells treated with oseltamivir. (DOCX 710 kb)

## Data Availability

All data used during the current study are available from the corresponding author on reasonable request.

## Abbreviations

CMI: Cumulative mutation index
Fluc: Firefly luciferase
RdRp: RNA-dependent RNA polymerase
Rluc: Renilla luciferase
