# Supplementary material for: Naturally occurring mutations in PB1 affect influenza A virus replication fidelity, virulence, and adaptability
Source: J Biomed Sci. 2019 Jul 31;26:55. doi: 10.1186/s12929-019-0547-4 (PMC6668090; doi:10.1186/s12929-019-0547-4)
Supplement: Supplementary file 1 — : Table S1 Epidemiological study on oseltamivir-resistant mutations in NA gene of human influenza A/H1N1 viruses using sequences deposited in NCBI influenza database. Figure S1 Evolution analysis of PB1–216 in influenza A/H3N2 shows serine-to-glycine point mutation at PB1–216 occurred in 1993. Figure S2 Schematic Diagram of influenza artificial genome containing dual-luciferase RT2AF for measuring RdRp fidelity during influenza virus replication. In the influenza virus-infected and RT2AF-transfected HEK cells, PolI starts to transcribe RT2AF as negative-strand viral RNA, which initiates self-replication of RT2AF reporter. The purpose of this reporter is to not only normalize total replication capability with the first Rluc activities but also evaluate the mutational potential that result in expression of the downstream Fluc activities. The Rluc activity reflects influenza replication levels. The Fluc activity measures the events in which RdRp repaired the engineered stop codon between the Rluc and Fluc reporters. The replication-driven Fluc activity thus represents the mutation potential of the virus. We calculated CMI based on the ratio of Fluc/Rluc that serves as an arbitrary measure of the number of mutation events occurring during virus replication and/or viral transcription. Figure S3 Epidemiological survey of residue substitution at PB1–43 and PB1–216 of influenza A viruses. Residues at PB1–43 of A/H5N1, H3N2 and H1N1 viruses and residues at PB1–216 of A/H1N1 viruses were examined using data derived from the Influenza Virus Database (GenBank) deposited prior to October 31, 2017. Figure S4 Effects of the PB1-S216G mutation on influenza A/H1N1 virus replication capability and adaptability in cells treated with oseltamivir. (DOCX 710 kb) [file 12929_2019_547_MOESM1_ESM.docx]

**Additional File**

**
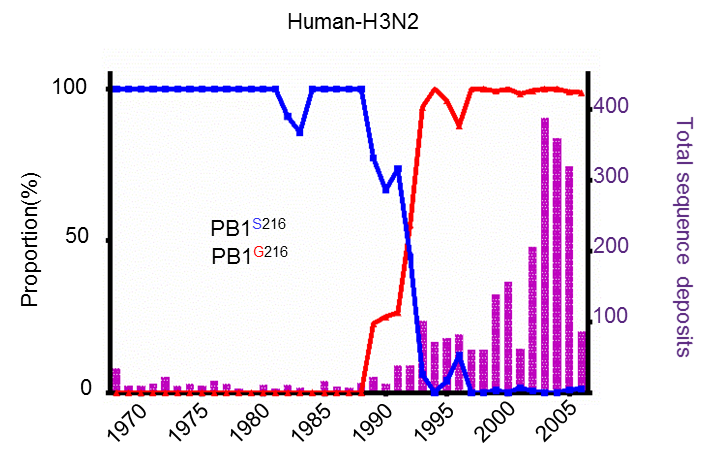
**

**Figure S1.** Evolution analysis of PB1-216 in influenza A/H3N2 shows serine-to-glycine point mutation at PB1-216 occurred in 1993. Residue substitution at PB1-216 of A/H3N2 was analyzed using data from the Influenza Virus Database (GenBank) that were deposited prior to August 31, 2016. Blue and red lines represent the proportion of serine and glycine residues at PB1-216, respectively. Purple bars represent number of full-length PB1 sequences submitted to the Database in the year indicated.


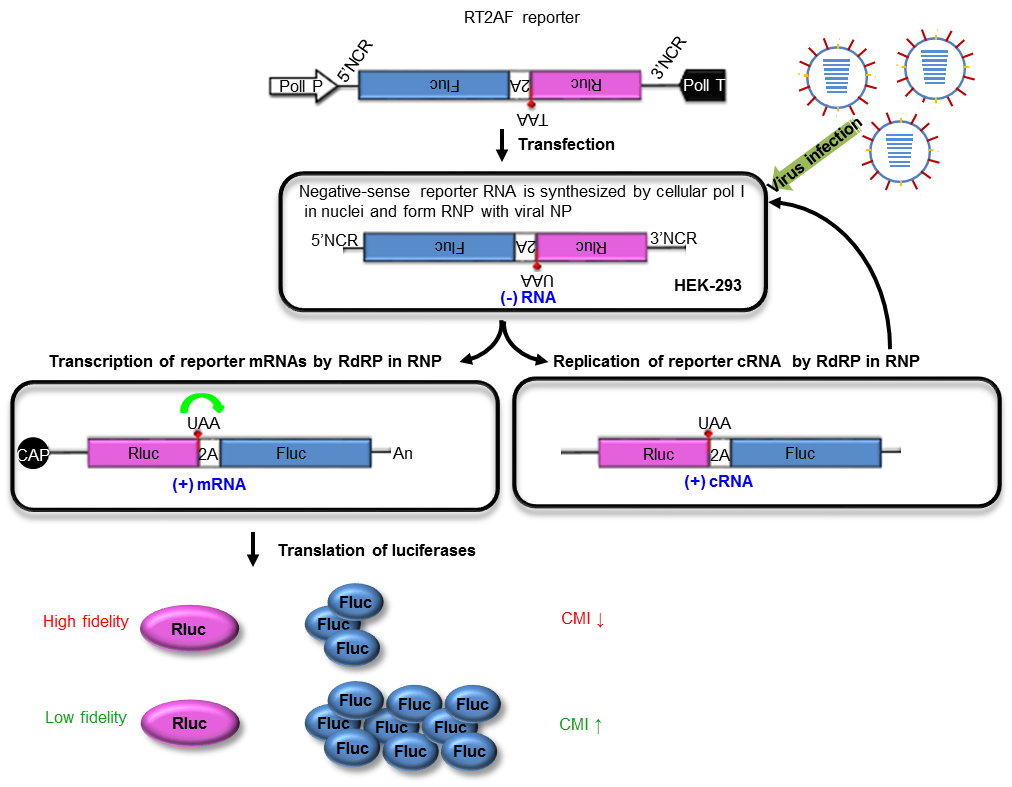


**Figure S2.** Schematic Diagram of influenza artificial genome containing dual-luciferase RT2AF for measuring RdRp fidelity during influenza virus replication. In the influenza virus-infected and RT2AF-transfected HEK cells, *PolI* starts to transcribe RT2AF as negative-strand viral RNA, which initiates self-replication of RT2AF reporter. The purpose of this reporter is to not only normalize total replication capability with the first Rluc activities but also evaluate the mutational potential that result in expression of the downstream Fluc activities. The Rluc activity reflects influenza replication levels. The Fluc activity measures the events in which RdRp repaired the engineered stop codon between the Rluc and Fluc reporters. The replication-driven Fluc activity thus represents the mutation potential of the virus. We calculated CMI based on the ratio of Fluc/Rluc that serves as an arbitrary measure of the number of mutation events occurring during virus replication and/or viral transcription.

**
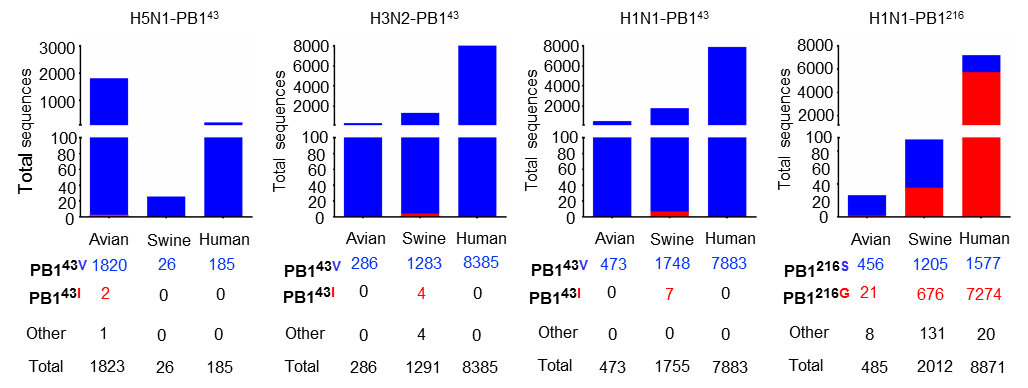
**

**Figure S3.** Epidemiological survey of residue substitution at PB1-43 and PB1-216 of influenza A viruses. Residues at PB1-43 of A/H5N1, H3N2 and H1N1 viruses and residues at PB1-216 of A/H1N1 viruses were examined using data derived from the Influenza Virus Database (GenBank) deposited prior to October 31, 2017.


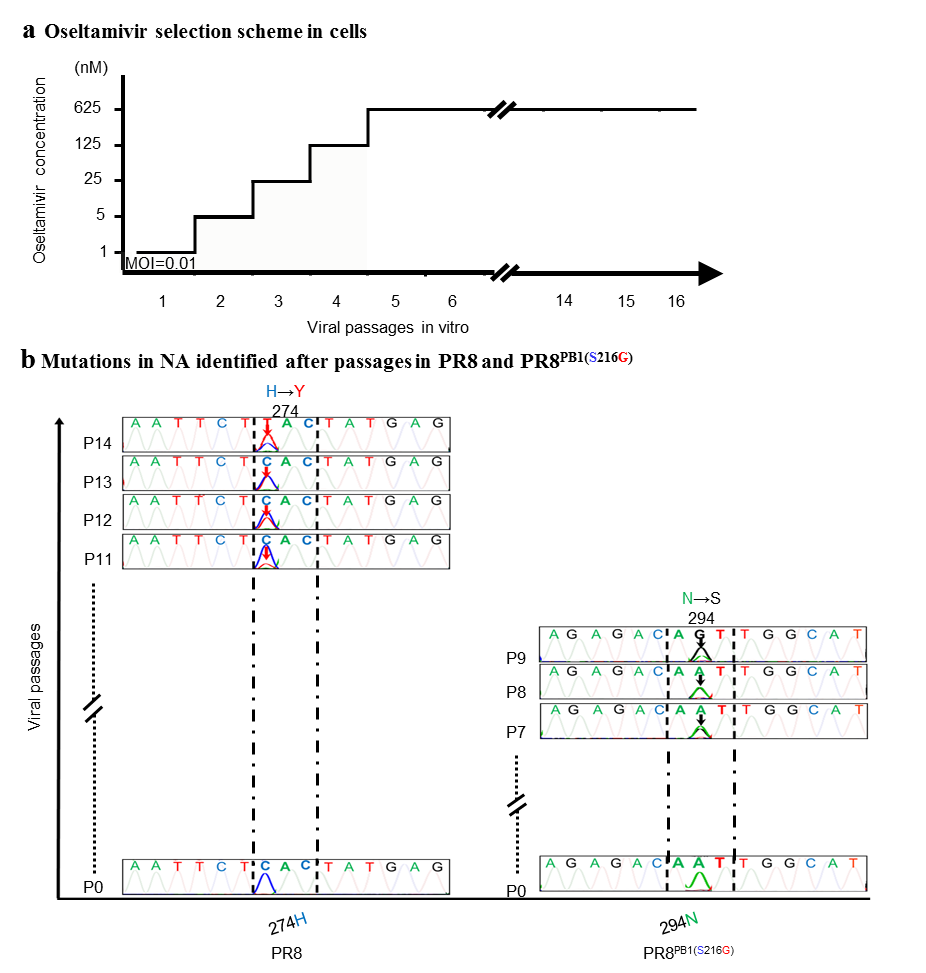


**Figure S4** Effects of the PB1-S216G mutation on influenza A/H1N1 virus replication capability and adaptability in cells treated with oseltamivir. (**a**) Cell-based oseltamivir selection scheme. Oseltamivir concentrations were increased 5-fold per passage, from 1 nM at passage 1 to 625 nM, approximately 0.25- (4.2nM) to 148-fold IC50, at passage 5 and thereafter. (**b**) The representative data of two independent experiments with similar results was shown, in which mutations in NA after passages in PR8^PB1(S216G)^ and PR8 were identified by direct Sanger sequencing of viral cDNA. The NA^N294S^ and NA^H274Y^ mutation were first identified at passage 7 (P7) from PR8^PB1(S216G)^ and passage 11 (P11) from PR8, respectively.

**Table S1.** Epidemiological study on oseltamivir-resistant mutations in NA gene of human influenza A/H1N1 viruses using sequences deposited in NCBI influenza database.

**
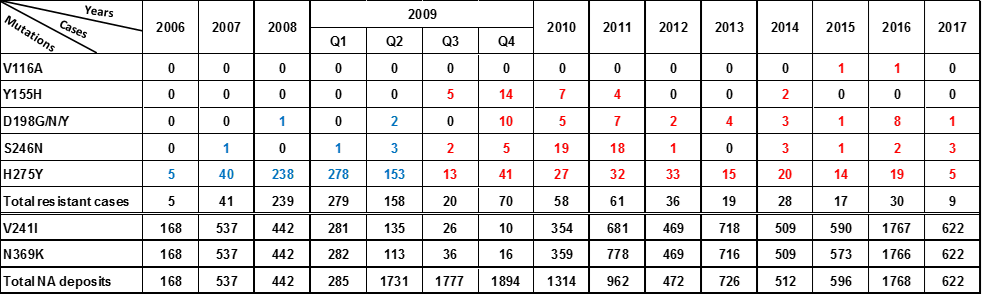
**

Complete full-length *NA* sequences for PB1-216S viruses (blue) and PB1-216G viruses (red) were retrieved from the Influenza Virus Database (GenBank). The sequences were aligned and examined to identify NA point mutations conferring oseltamivir resistance and permissive secondary mutations in NA (V241I and N369K).
